# Validation of an mHealth adoption questionnaire for osteoporosis management in Iranian older adults at risk

**DOI:** 10.1007/s11657-026-01741-6

**Published:** 2026-07-22

**Authors:** Golaleh Karbasi, Siti Anom Ahmad, Ghobad Moradi, Mahmoud Danaee, Nur Hafidah Ishak, Puvaneswaran Kunasekaran, Mohd Nazim Mohtar

**Affiliations:** 1 Malaysian Research Institute On Ageing (MyAgeing®), Universiti Putra Malaysia, Serdang, 43400, Selangor Malaysia; 2 Department of Electrical and Electronics Engineering, Faculty of Engineering, Universiti Putra Malaysia, Serdang, 43400 Selangor Malaysia; 3 Social Determinants of Health Research Center, Research Institute for Health Development, Kurdistan University of Medical Sciences, Sanandaj, Iran; 4 Department of Social and Preventive Medicine, Faculty of Medicine, University of Malaya, Kuala Lumpur, Malaysia; 5 Department of Psychiatry, Faculty of Medicine and Health Sciences, Universiti Putra Malaysia, Serdang, 43400 Selangor Malaysia; 6 Department of Social and Development Sciences, Faculty of Human Ecology, Universiti Putra Malaysia, Serdang, 43400 Selangor Malaysia

**Keywords:** mHealth adoption, Osteoporosis, Questionnaire validation, Older adults, Digital literacy, Self-efficacy

## Abstract

**Background:**

With the increasing prevalence of osteoporosis among older adults and the growing need for effective self-management strategies, mobile health (mHealth) technologies may play an important role in supporting osteoporosis management. This study aimed to adapt and validate a culturally appropriate Persian questionnaire to assess mHealth adoption for osteoporosis management among Iranian adults aged ≥50 years, regardless of osteoporosis diagnosis status.

**Methods:**

A questionnaire was adapted from established theoretical frameworks, including the Unified Theory of Acceptance and Use of Technology (UTAUT), the Health Belief Model (HBM), self-efficacy, digital literacy, and technology anxiety. The instrument underwent cross-cultural adaptation and psychometric evaluation in two phases: (1) content validation and cognitive testing, and (2) construct validation using exploratory factor analysis (EFA) and confirmatory factor analysis (CFA) with SmartPLS version 4.

**Results:**

The final instrument consisted of 54 items after removing poorly performing indicators. The measurement model demonstrated satisfactory psychometric properties, with average variance extracted (AVE) values ranging from 0.608 to 0.833 and composite reliability (CR) values between 0.866 and 0.950. Discriminant validity was supported by heterotrait–monotrait ratio (HTMT) values below the recommended threshold. Performance expectancy, self-efficacy, digital literacy, and perceived severity emerged as key determinants of mHealth adoption among older adults.

**Conclusion:**

This study developed a culturally adapted Persian questionnaire with strong reliability and validity for assessing mHealth adoption in osteoporosis management among older adults. The instrument may help researchers, clinicians, and policymakers identify barriers to digital health engagement and design targeted interventions to improve mHealth adoption in ageing populations.

***Summary*:**

The study designed and validated a culturally appropriate questionnaire to assess the acceptance of mHealth for osteoporosis management in Iranian adults over 50. Key predictors included performance expectancy, self-efficacy, and digital literacy. The findings support implementing tailored interventions to increase mHealth acceptance among older adults in resource-limited settings.

**Supplementary information:**

The online version contains supplementary material available at 10.1007/s11657-026-01741-6.

## Introduction

With advancements in medical research, the global population of older adults is increasing rapidly, particularly in low- and middle-income regions, where chronic diseases pose a growing burden [1–3].These demographic shifts are placing increasing pressure on healthcare systems and highlighting the need for systemic healthcare reforms, particularly in resource-limited settings. In recent years, healthcare has undergone many changes, from the *classic* model in which information is received from healthcare professionals as the "gatekeepers" of patient data [4] to a more collaborative model that has transformed between professionals and users, with patients participating in decision-making [5]. While health professionals initially had doubts about the accuracy and reliability of health-related technologies [6], growing familiarity with these technologies has highlighted their potential to facilitate access to and transfer of medical knowledge [6]. In this context, internet-based platforms have emerged as key channels for disseminating health information, leading to the development of the concept of “eHealth.”

eHealth refers to the use of information and communication technologies -including internet services, mobile communications, and voice systems—to support health education, healthcare delivery, and disease management [7]. A specialized subset of eHealth, mobile health, focuses on delivering medical care via smartphones and tablets, collecting and monitoring health data, and facilitating individual health practices. The COVID-19 pandemic further accelerated the global adoption of digital technologies [8], highlighting the role of mHealth in chronic disease management [9], and remote patient support [10, 11]. Older adults have been identified as the subpopulation that could benefit most from mHealth services [12]. Inaccessibility to healthcare- due to cost, staffing shortages, or mobility issues- has contributed to underutilization of healthcare services among older adults [13]. At the same time, Smartphone adoption among adults aged 55–91 has increased substantially, reaching approximately 40–68% in 2019, suggesting that mHealth tools can help bridge this gap [14, 15]. Consequently, mHealth has emerged as a promising strategy to support healthy aging and self-management [15, 16].

However, the adoption of technologies among older adults remains challenging. Previous studies indicate that up to 43% of adults aged 70 years and older discontinue using mobile health applications within the first two weeks [14], often due to poor usability, which has been cited as a major barrier. Usability is considered a vital factor influencing the adoption of mHealth by the elderly, which is defined as “the extent to which specified users can use a system to achieve specified goals with effectiveness, efficiency, and satisfaction within a specified context of use” [14].

Osteoporosis, the fourth most common chronic disease in older adults, affects approximately 200 million people worldwide and imposes a significant personal and economic burden [17]. In Iran, the age-standardized prevalence among those aged ≥ 60 years is estimated at 24.6% in men and 62.7% in women [18]. Limited disease-specific knowledge and inadequate support for osteoporosis self-management remain important global challenges that highlight the potential role of digital health solutions in bridging this gap.

Originally defined as “wireless eHealth” in the early 2000 s, mHealth has become a widely adopted model for public and professional healthcare [19]. Its flexibility- particularly bring-your-own-device (BYOD) models- has reduced barriers to use, while also raising concerns about funding and data privacy. Despite these challenges, mHealth represents one of the fastest-growing health tech categories [19]. Previous studies have demonstrated the potential of mHealth interventions in supporting chronic disease management, improving patient engagement, and facilitating self-monitoring [20, 21]. According to the World Health Organization, approximately 90% of people worldwide have access to wireless and mobile devices [22]. With over 350,000 health apps available, digital health platforms are increasingly influencing healthcare delivery [23]. This rapid expansion has encouraged healthcare systems to reconsider traditional models of care, particularly for the management of non-communicable diseases [24, 25]. Nevertheless, the use of mHealth technologies among older adults remains relatively low in countries such as Iran [26]. Adoption is often shaped by factors including perceived ease of use, usefulness, social influence, self-efficacy, attitude, and privacy risks [27]. Understanding these determinants is essential for improving the integration of digital health technologies into healthcare systems and enhancing their acceptance among older adults [28].

Accordingly, this study aimed to develop and validate a culturally adapted questionnaire to assess factors influencing the acceptance of mobile health technologies for osteoporosis management among Iranian adults aged ≥ 50 years- encompassing the general elderly population, both those diagnosed with osteoporosis and those at risk. Guided by established technology-acceptance frameworks, an initial questionnaire was developed in English using validated instruments as references, translated into Persian, and culturally adapted for the Iranian context. To our knowledge, only a limited number of studies have developed culturally adapted instruments to assess mHealth acceptance in the context of osteoporosis, and most existing tools have primarily focused on usability rather than broader behavioral determinants. Importantly, this study introduces a culturally adapted and theory-driven instrument to assess mHealth acceptance while also investigating attitudes toward osteoporosis management among older adults in Iran.

## Materials and methods

Cross-cultural adaptation and validation of a 54-item questionnaire assessing factors associated with the intention of adults aged ≥ 50 years to use mHealth technology for osteoporosis management in the Iranian context. The target population consisted of Iranian adults living in the community aged ≥ 50 years. A definitive diagnosis of osteoporosis was not required for participation in the study, as this age group is at the highest risk of developing osteoporosis. The study was conducted in two phases: (1) translation into Persian and evaluation of face and content validity, and (2) psychometric assessment, including construct validity and reliability analysis. Ethical approval was obtained from the Research Ethics Committee of the Endocrine and Metabolism Research Institute, Tehran University of Medical Sciences (ID: IR.TUMS.EMRI.REC.1403.180).

### Research instrument

The questionnaire, adapted from the scientific literature and based on the proposed study models, was divided into four sections: demographics, independent variables, mediating variables, and dependent variables. The variables were measured using a five-point Likert scale ranging from 1 (strongly disagree) to 5 (strongly agree), which is simple for respondents and suitable for survey studies [29]. Higher scores indicate stronger agreement and a greater level of the corresponding construct. For constructs such as performance expectancy, self-efficacy, digital literacy, and adoption intention, higher scores indicate greater readiness to adopt mobile health. In contrast, higher technology anxiety scores reflect greater concern about using technology. Possible subscale scores range from 1 to 5.

### Phase I: Content validity and translation

Face and content validity: Six experts (Table 1)—one from Malaysia and five from Iran -دwere selected based on their expertise in aging and healthcare research and assessed the questionnaire. The relevance and clarity of each item were assessed using a four-point scale (1 = not relevant/unclear to 4 = very relevant/very clear). To quantify agreement, the Content Validity Index (CVI) and modified Kappa coefficient were calculated, following Pollitt et al. (2007) and Litwin (1995). Items with CVI ≥ 0.80 and Kappa ≥ 0.70 were retained.

**Table 1:** List of panel of experts

No	Expert	Area of Expertise	Organization	Nationality
1	Dr. Sharafi	Associate Professor of Epidemiology	Elderly Health Research Center	Iran
2	Dr. Saeed Emadi	University Professor, Specialist Board in Diseases of the Elderly	-	Iran
3	Dr. Rahmat Dapari	Doctor of Public Health	Faculty of Medicine and Health Sciences, UPM	Malaysia
4	Dr. Jamileh Amirzadeh	Associate Professor in Medical Gerontology	Urmia University of Medical Sciences	Iran
5	Dr. Ahmad Delbari	Associate Professor, Geriatrician	Karolinska University, Sweden	Iran
6	Dr. Mahmood Danaee	Department of Social and Preventive Medicine, Faculty of Medicine	University of Malaya	Iran

**Fig. 1:**
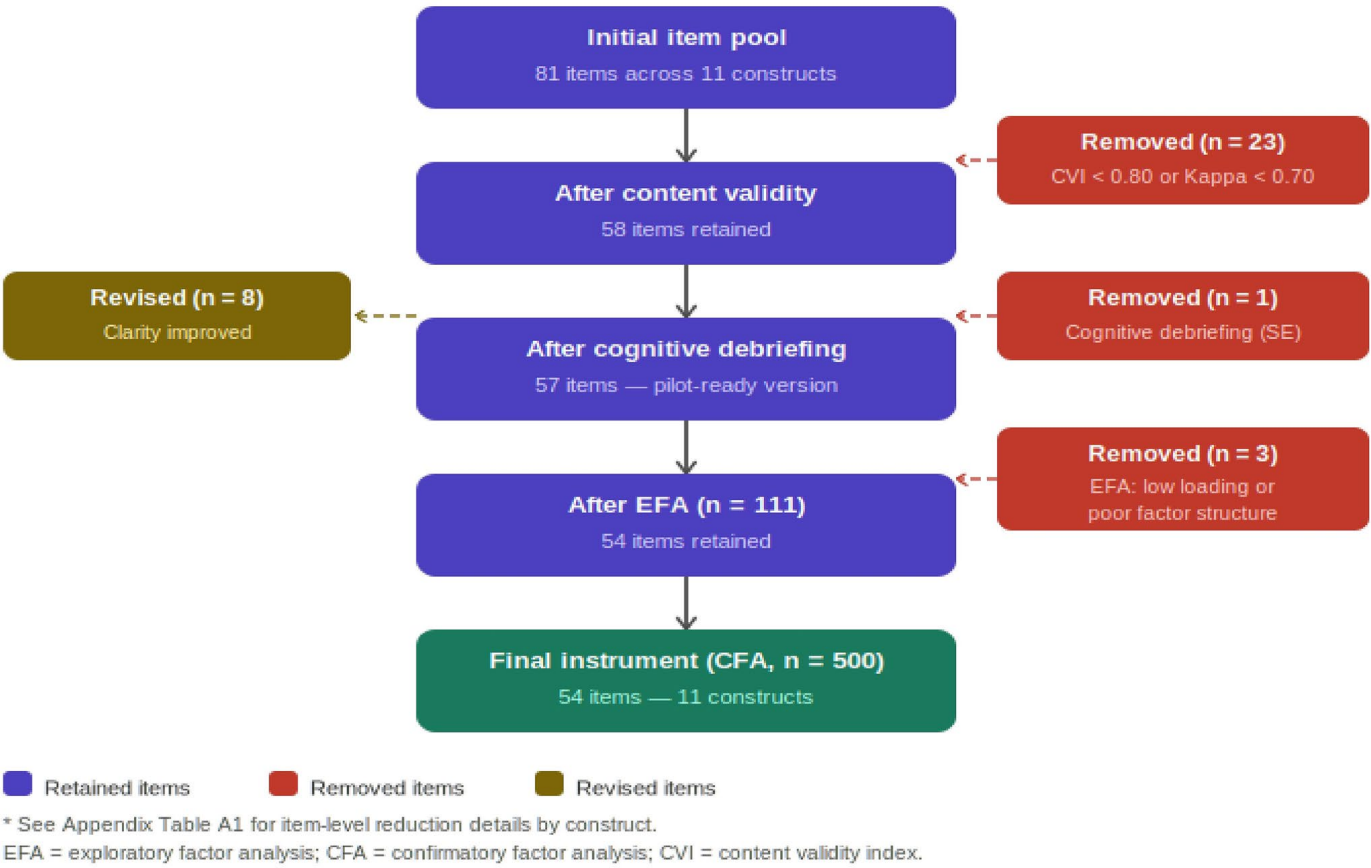
Flow diagram of questionnaire development and item reduction

The questionnaire's face validity was assessed through participant feedback during the cognitive debriefing stage. Discriminant validity was later evaluated during the construct validation phase using the heterotrait–monotrait (HTMT) ratio. Expert and participant suggestions led to item refinement. The core research team, including specialists in gerontology, epidemiology, geriatrics, and public health, revised the tool, resulting in a 57-item version for psychometric evaluation, which was further reduced to a final 54-item questionnaire after exploratory factor analysis (EFA). The overall process of questionnaire development and item reduction is illustrated in Fig. 1 (Appendix C, Table C).

Translation and cross-cultural adaptation. The translation and cross-cultural adaptation followed specific guidelines [30]. The process involved forward translation from English to Persian by two translators, followed by review and adaptation by the core research team (six faculty members from the Faculty of Gerontology, Medicine, and Health), resulting in a single Persian version. Then, reverse translation from Persian to English was performed, followed by re-evaluation for consistency by the core research team. Cognitive debriefing with potential participants was conducted to assess comprehension difficulties and the time required to complete the questionnaire.

### Phase II: Psychometric assessment

Psychometric evaluation of the Persian version of the questionnaire was conducted, including exploratory factor analysis (EFA) and reliability assessment. Based on the results of EFA in the first data set, CFA was conducted on the second data set (*N* = 500) using Smart-PLS Version 4. Written informed consent was obtained from participants, and data were collected and tested for construct validity and reliability.

### Data collection

Data were collected from Iranian adults aged ≥ 50 years between February and April 2023 using both online and in-person questionnaires. Participants were informed about the study objectives, provided written informed consent, responses were recorded anonymously using a 5-point Likert scale. Inclusion criteria required that participants were aged ≥ 50 years, regardless of osteoporosis diagnosis status, and cognitively and physically able to complete the questionnaire. Individuals with severe medical conditions that could preclude participation were excluded. Ethical approval was obtained from the Endocrine and Metabolism Research Institute, Tehran University of Medical Sciences (ID: IR.TUMS.EMRI.REC.1403.180).

### Statistical analysis

Sample adequacy was assessed using the Kaiser–Meyer–Olkin (KMO) test and Bartlett’s test of sphericity, with acceptable thresholds of KMO > 0.60 and p < 0.05. The data were divided into two subsets. EFA was conducted on the first subset (*n* = 111) to identify the factor structure of the 57-item questionnaire using principal component analysis (PCA) with Varimax rotation. Factor retention was based on three criteria applied simultaneously: (1) eigenvalues greater than 1.0 (Kaiser criterion), (2) inspection of the scree plot, and (3) conceptual interpretability of the extracted factors. Items were removed if their factor loading was below 0.50 or if their communality was below 0.30. No threshold of 0.35 was applied at any stage; all reported removals were based on the 0.50 loading criterion. All statistical analyses were performed using SPSS version 29 software. The second dataset (*N* = 500) was used for CFA to assess the dimensions.

## Results

### Phase I

Content and Face validity. An initial review by the research team showed strong content validity across most items (CVI = 0.70–0.79, Kappa = 0.60–0.74), except for 14 that failed to meet threshold values and were removed. These covered EE (2 items), FC (2), SI (1), PSU (1), TA (4), DL (1), and SE (4). Additionally, nine items presented clarity concerns (CVI = 0.61–1.00, Kappa = 0.42–1.00); among these, 1 was removed, and eight were revised for clarity based on expert feedback. Full item-level details are presented in Appendix B.

Translation and Cognitive debriefing. The questionnaire was translated using forward and backward translation. Two native Persian speakers -an English professor and a public health expert—prepared the initial versions. The core research team, consisting of six faculty members in gerontology, medicine, and public health, evaluated the draft to reach consensus. The Persian version was then back-translated by two independent bilingual public health experts to ensure conceptual alignment with the original. Finally, the questionnaire was administered to 10 older adult volunteers for cognitive testing, assessing clarity, completion time, and comprehension. All items were well understood, except for one in the effort expectancy (EE) construct, which was removed due to misunderstanding (Appendix A, Table A: Full Questionnaire).

### Phase II (construct validity)

Sociodemographic Characteristics. A total of 111 respondents completed the questionnaire. The majority were male (62.16%), and most participants were non-native residents of Tehran (84.68%). The largest age group was 71–75 years **(**Table 2**).**

**Table 2:** Demographic Characteristics of Respondents for EFA and CFA Sample

Demographic Characteristics	EFA Sample (*n* = 111)	CFA Sample (*n *= 500)
Gender		
Male	69(62.16%)	232 (46.4%)
Female	42 (37.84%)	268 (53.6%)
Place of Birth		
Tehran	13(11.71%)	160 (32%)
Other Cities (Specify)	94(84.68%)	328 (65.6%)
Age Group		
50–55	11(9.91%)	153 (30.6%)
56–60	4(3.60%)	127 (25.4%)
61–65	1(0.90%)	84 (16.8%)
66–70	1(0.90%)	55 (11%)
71–75	37(33.33%)	57 (11.4%)
76–80	30(27.03%)	13 (2.6%)
81–86	18(16.22%)	5 (1%)
86 and above	9(8.11%)	3 (0.6%)
Marital Status		
Single	8(7.21%)	43 (8.6%)
Married	101(90.99%)	423 (84.6%)
Divorced	2(1.80%)	33 (6.6%)
Education Level		
Illiterate	18 (16.22%)	79 (15.8%)
Primary/Intermediate	12(10.81%)	49 (9.8%)
Secondary/Diploma	29(26.13%)	162 (32.4%)
Associate Degree	10(9.01%)	43 (8.6%)
Bachelor’s Degree	26(23.42%)	116 (23.2%)
Master's/PhD	16(14.41%)	48 (9.6%)
Occupation		
Retired	-	189 (37.8%)
Unemployed	49(44.14%)	10 (2%)
Government Employee	11(9.91%)	50 (10%)
Private Company Employee	6(5.41%)	33 (6.6%)
Self-Employed	12(10.81%)	73 (14.6%)
Homemaker	32(28.83%)	143 (28.6%)
Other (Specify)	1(0.90%)	-
Monthly Income (IRR)		
Less than 50 million	33(29.73%)	62 (12.4%)
50 to 100 million	25(22.52%)	95 (19%)
100 to 150 million	23(20.72%)	140 (28%)
150 to 200 million	14(12.61%)	105 (21%)
200 to 250 million	6(5.41%)	50 (10%)
More than 250 million	7(6.31%)	27 (5.4%)

*Percentages are calculated based on the total respondents in each sample group. *

Exploratory factor analysis. EFA was conducted on the first dataset (*n *= 111) using principal component analysis (PCA) with Varimax rotation. Factor retention for components (UTAUT, HBM, SE, DL, and TA) was based on eigenvalues greater than 1.0 (Kaiser criterion), inspection of the scree plot, and conceptual interpretability. Items with communalities below 0.30 or factor loadings below 0.50 were removed.

UTAUT Component. EFA was conducted on 20 items related to UTAUT using Varimax rotation. KMO value of 0.881 indicated excellent sampling adequacy, and Bartlett’s test was significant (χ^2^ (190) = 1894.694, *p* < 0.001). All communalities exceeded 0.30, and factor loadings were above 0.50. Five components were extracted with eigenvalues > 1, representing PE (45.58% variance), IA (11.77%), SI (9.11%), EE (6.65%), and FC (5.37%), as shown in Table 3.

**Table 3:** Principal component loadings of measurement items based on Varimax rotation

Construct	Component	Item	Loading	Eigenvalue	Variance (%)
UTAUT	C1	PE2	0.848	9.117	45.583
		PE5	0.842		
		PE1	0.840		
		PE4	0.820		
		PE3	0.773		
	C2	IA2	0.865	2.354	11.770
		IA4	0.862		
		IA3	0.846		
		IA1	0.815		
		IA5	0.741		
	C3	SI2	0.807	1.822	9.108
		SI4	0.803		
		SI1	0.803		
		SI3	0.802		
	C4	EE2	0.818	1.330	6.510
		EE1	0.796		
		EE3	0.747		
	C5	FC1	0.837	1.074	5.369
		FC2	0.684		
		FC3	0.624		
HBM	C6	PSU3	0.891	3.430	38.107
		PSU4	0.837		
		PSU2	0.832		
		PSU1	0.790		
	C7	PSE2	0.888	2.165	24.058
		PSE3	0.874		
		PSE1	0.728		
		PSE4	0.597		
		PSE5	0.487		
SE	C8	SE2	0.942	2.581	57.019
		SE1	0.879		
		SE3	0.796		
		SE4	0.707		
DL	C9	DL8	0.926	6.508	72.315
		DL7	0.922		
		DL5	0.873		
		DL6	0.870		
		DL11	0.861		
		DL2	0.855		
		DL9	0.846		
		DL10	0.824		
		DL1	0.645		
TA	C10	TA2	0.902	3.440	49.147
		TA4	0.815		
		TA3	0.791		
		TA1	0.785		
	C11	TA7	0.914	1.320	18.864
		TA6	0.854		
		TA5	0.482		

Extraction method: principal component analysis; rotation method: Varimax with Kaiser normalization. Only loadings ≥ 0.30 are shown

HBM Component. EFA using Varimax rotation was applied to 9 items assessing HBM. The KMO measure was 0.742, and Bartlett’s test was significant (χ^2^ (36) = 462.334, p < 0.001), supporting factorability. All communalities and loadings were acceptable (> 0.30 and > 0.50, respectively). Two components emerged with eigenvalues > 1: PSU (38.11% variance) and PSE (24.06%), detailed in Table 3**.**

Self-Efficacy Component. EFA was performed on 5 SE items using Varimax rotation. Sampling adequacy was confirmed with a KMO value of 0.731, and Bartlett’s test reached significance (χ^2^ (10) = 248.694, p < 0.001). Communalities were above 0.30, and all loadings exceeded 0.50 except for SE5, which showed a low loading (< 0.30) and was removed. The final one-factor solution explained 57.02% of the variance in Table 3.

Digital Literacy Component. EFA was conducted on 11 items related to digital literacy, with a KMO value of 0.899 and a significant Bartlett’s test (χ^2^ (55) = 1295.326, p < 0.001). Initial analysis suggested two components; however, items DL3 and DL4 formed a separate factor, compromising unidimensionality. After removing DL3 and DL4 due to poor factor structure, a unidimensional solution with nine items was retained, explaining 72.32% of the variance (Table 3).

Technology Anxiety Component. Seven items were analyzed using EFA with Varimax rotation. The KMO value was 0.734, and Bartlett’s test was significant (χ^2^ (21) = 352.856, p < 0.001). All communalities and loadings were acceptable. EFA identified two factors (TA1–TA4 and TA5–TA7 with eigenvalues > 1, explaining 49.15% and 18.86% of the variance, respectively. These represented two subdimensions of technology anxiety, forming a second-order component. A repeated indicator approach was initially explored in SmartPLS to model this second-order structure (Table 3). However, a confirmatory factor analysis conducted using SmartPLS on the full sample (*n *= 500) indicated significant overlap between these sub-dimensions and insufficient discriminant validity. Therefore, they were combined and considered as a single technology anxiety construct in the final measurement model.

Confirmatory Factor Analysis was conducted on the second dataset (*n* = 500) using SmartPLS v4, based on the reflective nature of all variables. Convergent validity was assessed through indicator loadings, average variance extracted (AVE), and internal consistency via composite reliability (CR) and Cronbach’s alpha. According to Hair et al. (2010) and Fornell & Larcker (1981), indicators should ideally show outer loadings ≥ 0.708; however, loadings > 0.50 are acceptable in exploratory contexts with AVE ≥ 0.50, and CR ≥ 0.70. In this study, all item loadings exceeded 0.5 (range: 0.641–0.928) except for HS5 (0.343), which was excluded. AVE ranged from 0.608 to 0.833, CR from 0.866 to 0.950, and Cronbach’s alpha from 0.781 to 0.938, indicating strong reliability and convergent validity.

Discriminant validity was evaluated using the Fornell-Larcker criterion, heterotrait–monotrait ratio (HTMT), and cross-loadings. Following Hair Jr. et al. (2010), HTMT values below 0.90 were considered acceptable. All constructs met this threshold, confirming discriminant validity (Tables 4 and 5). Although the HTMT value between effort expectancy and facilitating conditions approached the threshold (0.829), it remained below the accepted criterion of 0.90, confirming adequate discriminant validity between these constructs.

**Table 4:** The result of convergent validity**:** Factor Loadings, Reliability, and Average Variance Extracted

Construct	Item	Loading Factor		Cronbach’s alpha	Composite reliability	Average variance extracted (AVE)
		Initial model	Modified model			
DL	DL1	0.722	0.722	0.938	0.948	0.672
	DL10	0.806	0.806			
	DL11	0.829	0.829			
	DL2	0.814	0.814			
	DL5	0.813	0.813			
	DL6	0.750	0.750			
	DL7	0.888	0.888			
	DL8	0.892	0.892			
	DL9	0.847	0.847			
EE	EE1	0.923	0.923	0.899	0.937	0.833
	EE2	0.928	0.928			
	EE3	0.886	0.886			
FC	FC1	0.821	0.821	0.781	0.872	0.695
	FC2	0.862	0.862			
	FC3	0.818	0.818			
HS	HS1	0.822	0.822	0.794	0.866	0.618
	HS2	0.788	0.788			
	HS3	0.811	0.811			
	HS4	0.720	0.720			
	HS5	0.343	Del			
IA	IA1	0.844	0.844	0.926	0.944	0.773
	IA2	0.911	0.912			
	IA3	0.900	0.900			
	IA4	0.902	0.901			
	IA5	0.835	0.835			
PE	PE1	0.887	0.887	0.934	0.95	0.791
	PE2	0.901	0.901			
	PE3	0.872	0.872			
	PE4	0.888	0.888			
	PE5	0.900	0.900			
PSE	PSE1	0.785	0.785	0.837	0.885	0.608
	PSE2	0.905	0.905			
	PSE3	0.825	0.825			
	PSE4	0.718	0.717			
	PSE5	0.641	0.641	0.868	0.902	0.7
PSU	PSU1	0.684	0.684			
	PSU2	0.839	0.839			
	PSU3	0.912	0.912			
	PSU4	0.893	0.893			
SE	SE1	0.897	0.896	0.899	0.93	0.769
	SE2	0.928	0.928			
	SE3	0.846	0.846			
	SE4	0.833	0.833	0.892	0.926	0.757
SI	SI1	0.896	0.896			
	SI2	0.904	0.904			
	SI3	0.872	0.872			
	SI4	0.805	0.805			
TA	TA1	0.813	0.813	0.902	0.922	0.631
	TA2	0.869	0.869			
	TA3	0.813	0.813			
	TA4	0.854	0.854			
	TA5	0.705	0.705			
	TA6	0.794	0.794			
	TA7	0.696	0.696

**Table 5:** Heterotrait–Monotrait Ratio (HTMT) of Correlations for Discriminant Validity Assessment

	1	2	3	4	5	6	7	8	9	10	11
Technology anxiety											
Digital literacy	**0.513**										
Effort expectancy	0.519	**0.745**									
Facilitating conditions	0.425	0.705	**0.829**								
Health status	0.374	0.346	0.299	**0.244**							
Intention to adopt mHealth technology	0.464	0.661	0.676	0.669	**0.292**						
Perceived severity	0.102	0.107	0.087	0.099	0.14	**0.079**					
Perceived susceptibility	0.233	0.139	0.189	0.154	0.393	0.175	**0.329**				
Performance expectancy	0.321	0.587	0.714	0.67	0.192	0.647	0.131	**0.044**			
Self-efficacy	0.514	0.759	0.758	0.728	0.383	0.747	0.126	0.187	**0.653**		
Social influence	0.211	0.462	0.588	0.681	0.134	0.557	0.115	0.055	0.56	**0.55**	

HTMT values below 0.90 indicate adequate discriminant validity. All values meet the recommended threshold

## Discussion

Cultural and social diversity has influenced ageist attitudes rooted in traditional norms, limiting older adults’ access to health care [13]. To reduce such biases, cross-cultural adaptation of assessment tools is crucial when used in different linguistic and cultural contexts[31]. The culturally adapted Persian questionnaire developed in this study may facilitate the adoption of mHealth technologies for osteoporosis management and contribute to broader efforts aimed at improving inclusiveness in digital health [26].Across Southeast Asia, screening tools such as the OSTA have been localized for use in Malaysia and Thailand, while China has validated the OKT questionnaire [32, 33]. Globally, digital solutions such as Denmark’s “My Bones” program for patient self-care and in Iran, tools such as QUALEFFO-41 and PAQ-SCT for older populations have demonstrated high reliability (Cronbach’s alpha 0.90) [34, 35]. Given Iran’s high osteoporosis prevalence (24.6% of men and 62.7% of women over 60; Fahimfar et al. (2021)), the present study explored determinants of mHealth adoption to help inform strategies aimed at improving healthcare for older adults [9].This study developed and validated a 54-item questionnaire, making it suitable for future clinical and research use. Several items were excluded due to low validity (CVI = 0.61–1.00) and cultural mismatch. The removal of SE5, DL3, and DL4 during EFA due to weak loadings < 0.30 is consistent with procedures commonly applied in the validation of health measurement scales [35, 36], and emphasizes the need for cultural adaptation. This modification improves the questionnaire’s relevance for the Iranian context and supports future validation against established measures such as eHEALS [34], similar to adjustments made in a Malaysian mHealth study to ensure cultural relevance for a diverse population [37].

The five UTAUT constructs represent key dimensions for understanding mHealth adoption among Iranian seniors. Performance expectancy reflects the perceived usefulness of mHealth technologies [7, 33]. Intention to adopt indicates users’ readiness for regular technology use, consistent with the findings of Danish and Persian studies by Jensen et al. (2023) and Jeihooni et al. (2016), which emphasize behavioral intention to use technology.

Social influence indicates the role of family members and healthcare providers in shaping attitudes towards digital health, especially in collectivist cultures [38, 39]. However, in the context of health-related technologies, older adults may rely more strongly on perceived usefulness and personal confidence when deciding whether to adopt mHealth tools rather than on broader social expectations [39, 40]. Consequently, the greater influence of individual health needs and physician recommendations, compared to general social pressure, may explain the relatively limited role of social influence observed in the present findings. Effort expectancy emphasizes the importance of user-friendly design for seniors with limited digital skills, referring to ease of use as reported in studies by Liu et al. (2023) and Palas et al. (2022). Facilitating conditions represent the availability of technological infrastructure and support systems necessary for the sustained use of mHealth applications, as highlighted in previous studies [41–43].

The Health Belief Model component includes two key factors influencing mHealth acceptance. PSU, based on four items, reflected older adults’ beliefs about their susceptibility to osteoporosis complications and aligned with findings from Jeihooni, A.K. et al. (2016) and Ghelichkhani et al. (2021), supporting its role as a motivator in health engagement. PSE, captured through five items, assessed the perceived severity of osteoporosis outcomes. These findings are consistent with previous studies, including the Danish “My Bones” study (2023), reinforcing severity as a driver of mHealth adoption. The self-efficacy construct consists of four items reflecting older adults’ confidence in using mHealth. SE5 was excluded due to low loading (< 0.3). These findings are consistent with Ghelichkhani et al. (2021), who explored self-efficacy in osteoporosis prevention among Iranian adolescents, and with Abu Seman (2020), supporting the stability of this construct across populations [44]. A Malaysian study also reported that higher self-efficacy was associated with greater willingness to adopt mobile health services, reinforcing the stability of this construct across different populations [45]. The Digital Literacy component included nine items, refined by removing (DL3, DL4) to ensure consistency. These measured older adults’ ability to use mHealth tools effectively- an area similarly explored in Iranian studies on digital health literacy [46]. Norman and Skinner’s eHealth Literacy Model highlights how digital skills shape mHealth engagement [47]. Collectively, these findings underscore digital literacy as a critical determinant of mHealth adoption among older adults with varying levels of technological exposure.

The Technology Anxiety component was structured as a second-order construct with two subdimensions: general anxiety (TA1–TA4) and specific concerns, such as privacy and errors (TA5–TA7), consistent with those who identified technology anxiety as a multidimensional barrier to mHealth adoption [39]. Abdipour et al. (2024) validated the TechPH scale for Iranian older adults, showing that both willingness and anxiety shape mHealth adoption [48]. However, in the CFA conducted using SmartPLS (*n *= 500), the two subdimensions showed substantial overlap and did not demonstrate adequate discriminant validity. Therefore, they were combined and treated as a single construct in the final measurement model.

Interestingly, some constructs traditionally associated with technology adoption- such as perceived susceptibility, facilitating conditions, and technology anxiety -did not demonstrate strong associations with the external criterion measures used in this study. One possible explanation may relate to the characteristics of the study population. Older adults may rely more heavily on perceived usefulness of health technologies and their own confidence in using digital tools than on perceived vulnerability to disease outcomes when evaluating mHealth applications. [39, 40]. Regarding social influence in particular, although it is a valid predictor of technology acceptance in collectivist societies [39], its limited role in this study may be due to the lack of awareness of mHealth tools among Iranian older adults, where family members and healthcare providers have not yet actively promoted digital health solutions for osteoporosis management. This contrasts with findings for other chronic diseases, where social networks more actively drive technology acceptance[49].

In addition, the increasing penetration of smartphones and internet access in urban environments such as Tehran may reduce the perceived importance of facilitating conditions as barriers to technology use [42]. Furthermore, technology anxiety may diminish when individuals receive assistance from family members or healthcare professionals, which is common in collectivist cultures where intergenerational support influences technology engagement [7, 39]. These findings suggest that capability-related constructs -particularly digital literacy and self-efficacy-play a more central role in shaping readiness to engage with mHealth technologies among older adults in this context.

Confirmatory factor analysis (*n* = 500) validated the mHealth questionnaire for osteoporosis management among Iranian elderly adults, showing strong convergent and discriminant validity. Indicators met accepted thresholds [43] loadings ranged from 0.641–0.928 (except HS5, omitted), AVE from 0.608–0.833, CR from 0.866–0.950, and Cronbach’s alpha from 0.781–0.938. These results echo the usability findings in Malaysia [37]. HTMT values (0.044–0.829) confirmed discriminant validity, consistent with UTAUT validation in Iran [33]. Notably, digital literacy shows a collectivist high AVE (0.833) and moderate HTMT correlation with self-efficacy (0.759), reflecting its relevance for the predominantly non-native residents of Tehran in this sample (84.68%), supported by Santos et al. (2021). Overall, these findings highlight the need for culturally adapted and user-friendly mHealth tools and support their alignment with eHEALS frameworks for future validation (Table 5). To assess criterion validity, this study used the eHealth Literacy Scale (eHEALS) as a recognized standard due to its strong validity and reliability in measuring digital health literacy among older adults by Norman et al. (2010). Components showing significant positive correlations with mHealth included Performance Expectancy (PE1–PE5), Social Influence (SI1–SI4), Effort Expectancy (EE1–EE3), Self-Efficacy (SE1–SE4), and Digital Literacy (DL1, DL2, DL5–DL11)—25 of the 54 final items. These results align with prior findings from Norman et al. (2010) and Nematollahi & Eslami (2018), who validated health literacy scales across diverse populations, including Iranian populations. Meanwhile, IA, FC, and TA showed no significant correlation, likely due to their focus on behavioral and emotional aspects not directly addressed by eHEALS. For discriminative validity, this study found significant gender differences in PE, with male participants (62.16%) rating mHealth tools as more useful for managing osteoporosis -consistent with findings from Hoque et al. (2017) and Zhou et al. (2019). Age also played a role, as those aged 70–75 showed higher DL scores, possibly due to greater exposure to mHealth in urban Tehran, echoing Bahadori et al. (2024). In contrast, SI and TA did not vary across gender or residential status, suggesting that these factors may be relatively stable across demographic groups within this population. While the 54-item questionnaire provides a comprehensive assessment of factors influencing mHealth adoption, its length may limit routine use in clinical and public health settings. Nevertheless, comprehensive instruments are often necessary during the early stages of scale development to ensure adequate construct coverage and psychometric robustness [50]. A shortened version—potentially comprising the highest-loading items from each construct—could be developed and validated in future studies to facilitate rapid screening in orthopedic and geriatric care settings, while preserving the core psychometric properties of the full instrument.

### Clinical and policy implications

The validated questionnaire developed in this study provides clinicians and health policymakers with a practical tool to assess the determinants of mHealth adoption among older adults in osteoporosis management. Identifying factors such as digital literacy and self-efficacy can assist clinicians in designing targeted educational interventions to enhance older adults’ engagement with digital health technologies [39, 40]**.** Specifically, clinicians and program coordinators could administer this questionnaire prior to enrollment in a mHealth program to identify older adults at risk for low digital engagement and enable targeted pre-intervention support in digital literacy or self-efficacy education. At the policy level, this tool can inform national digital health strategies by mapping readiness gaps among older populations and guide the integration of digital health literacy initiatives into appropriate mHealth infrastructures, promoting user-friendly platforms and educational programs focused on osteoporosis prevention and bone health promotion [24, 47].

### Limitations and future research

This study has several limitations that should be acknowledged. To begin with, the cross-sectional design does not have the opportunity to develop causal connections among the examined constructs. Second, although the sample was drawn from Tehran, the capital city of Iran, with a relatively diverse population, the findings may not be generalizable to older adults living in less technologically developed or rural areas. Third, the self-reported measures may introduce response bias. Future research should employ longitudinal designs and include larger and more diverse populations to further validate the instrument. Additionally, this study focused on participants’ intention to adopt mHealth technologies rather than their actual usage behavior; therefore, future studies should examine whether these determinants translate into sustained real-world use of mHealth applications among older adults.

## Conclusion

The present study adapted and validated a culturally appropriate Persian instrument to assess mHealth adoption for osteoporosis management among Iranian adults aged ≥ 50 years. The 54-item questionnaire demonstrated strong validity and reliability, supported by CFA and established psychometric indicators, including AVE and CR. Performance expectancy, intention to adopt, self-efficacy, digital literacy, and perceived severity emerged as key determinants of mHealth adoption among older adults. These findings highlight the importance of user-centered and culturally tailored digital health interventions to support osteoporosis self-management in ageing populations.

## Supplementary information

Below is the link to the electronic supplementary material.

ESM 1 (DOCX 33.2 KB)

## Data Availability

Data available on reasonable request.

## Declarations

### Tip

An AI tool helped refine the draft by reducing words and supporting language editing and grammar refinement. Scientific content, data interpretation, final editing, and content decisions were made by the authors.

## References

[1] Guner H, CJUA (2020) It is Acarturk, The use and acceptance of ICT by senior citizens: a comparison of the technology acceptance model (TAM) for elderly and young adults. 19(2): 311–330

[2] Atun R (2015) Transitioning health systems for multimorbidity. Lancet 386(9995):721–722 26063473 10.1016/S0140-6736(14)62254-6

[3] Kanis JA et al (2021) SCOPE 2021: a new scorecard for osteoporosis in Europe. Arch Osteoporos 16(1):82 34080059 10.1007/s11657-020-00871-9 PMC8172408

[4] Eysenbach G, A.R. JomIr Jadad (2001) Evidence-based patient choice and consumer health informatics in the internet age. 3(2): e841 10.2196/jmir.3.2.e19 PMC1761898 11720961

[5] Tretiakov A, Whiddett D, I.J.I.j.o.m.i Hunter (2017) Knowledge management systems success in healthcare: Leadership matters*.* 97: 331–340 10.1016/j.ijmedinf.2016.11.004 27919392

[6] Altmann V, Gries M (2017) Factors influencing the usage intention of mHealth apps: An empirical study on the example of Sweden

[7] Akter S, D’Ambra J, Ray P (2013) Development and validation of an instrument to measure user perceived service quality of mHealth. Inf Manag 50(4):181–195

[8] Van Elburg FRT et al (2023) The intention to use mHealth applications among Dutch older adults prior and during the COVID pandemic. Front Public Health 11:1130570 37383259 10.3389/fpubh.2023.1130570 PMC10298165

[9] Changizi M, Kaveh MH (2017) Effectiveness of the mHealth technology in improvement of healthy behaviors in an elderly population—a systematic review. Mhealth 3:51 29430455 10.21037/mhealth.2017.08.06 PMC5803024

[10] Abbaspur-Behbahani S et al (2022) Application of mobile health to support the elderly during the COVID-19 outbreak: a systematic review. Health Policy Technol 11(1):100595 35018280 10.1016/j.hlpt.2022.100595 PMC8739352

[11] Alam MZ, Proteek S, Hoque I (2023) A systematic literature review on mHealth related research during the COVID-19 outbreak. Health Educ 123(1):19–40

[12] Yi JY et al (2018) Self-management of chronic conditions using mHealth interventions in Korea: a systematic review. Healthc Inform Res 24(3):187–197 30109152 10.4258/hir.2018.24.3.187 PMC6085202

[13] Liao Q et al (2021) Barriers to preventive care utilization among Hong Kong community-dwelling older people and their views on using financial incentives to improve preventive care utilization. Health Expect 24(4):1242–1253 33949749 10.1111/hex.13256 PMC8369124

[14] Wang Q et al (2022) Usability evaluation of mHealth apps for elderly individuals: a scoping review. BMC Med Inform Decis Mak 22(1):317 36461017 10.1186/s12911-022-02064-5 PMC9717549

[15] Liaw S-T et al (2019) Use of mHealth for promoting healthy ageing and supporting delivery of age-friendly care services: a systematic review. Int J Integr Care. 10.5334/ijic.s314731367204

[16] Elavsky S et al (2019) Mobile health interventions for physical activity, sedentary behavior, and sleep in adults aged 50 years and older: a systematic literature review. J Aging Phys Act 27(4):565–593 30507266 10.1123/japa.2017-0410

[17] Johnell O, J.J.O.i. Kanis (2006) An estimate of the worldwide prevalence and disability associated with osteoporotic fractures. 17:1726–1733 10.1007/s00198-006-0172-4 16983459

[18] Fahimfar N et al (2021) Prevalence of osteoporosis among the elderly population of Iran 16:1–10 10.1007/s11657-020-00872-8 33475880

[19] Brownlow L (2023) Boosting adoption of mobile health apps: An exploration of new human and technology drivers of adoption. Flinders University, College of Business, Government and Law

[20] Free C et al (2013) The effectiveness of mobile-health technology-based health behaviour change or disease management interventions for health care consumers: a systematic review. PLoS Med 10(1):e1001362 23349621 10.1371/journal.pmed.1001362 PMC3548655

[21] Dai M et al (2017) Willingness to use mobile health in glaucoma patients. Telemed J E Health 23(10):822–827 28418773 10.1089/tmj.2016.0254

[22] Organization, W.H. (2017) Global diffusion of eHealth: making universal health coverage achievable: report of the third global survey on eHealth. World Health Organization

[23] Mescher T et al (2025) Mobile health apps: guidance for evaluation and implementation by healthcare workers. Journal of Technology in Behavioral Science 10(2):224–235

[24] Feroz A, Kadir MM, Saleem S (2018) Health systems readiness for adopting mhealth interventions for addressing non-communicable diseases in low-and middle-income countries: a current debate. Glob Health Action 11(1):1496887 30040605 10.1080/16549716.2018.1496887 PMC6063338

[25] Mandari H, Yahaya M (2022) Examining factors influencing intention to use M-Health applications for promoting healthier life among smartphone users in Tanzania. J Int Technol Inf Manag 31(2):1–21

[26] Bashshur RL et al (2016) The empirical evidence for telemedicine interventions in mental disorders. Telemed J E Health 22(2):87–113 26624248 10.1089/tmj.2015.0206 PMC4744872

[27] Fan S, Jain RC, Kankanhalli MS (2024) A comprehensive picture of factors affecting user willingness to use mobile health applications. ACM Trans Comput Healthc 5(1):1–31

[28] Gurupur VP, Wan TT (2017) Challenges in implementing mHealth interventions: a technical perspective. Mhealth 3:32 28894742 10.21037/mhealth.2017.07.05 PMC5583043

[29] Bouranta N, Chitiris L, Paravantis J (2009) The relationship between internal and external service quality. Int J Contemp Hosp Manag 21(3):275–293

[30] Wild D et al (2005) Principles of good practice for the translation and cultural adaptation process for patient-reported outcomes (PRO) measures: report of the ISPOR task force for translation and cultural adaptation. Value Health 8(2):94–104 15804318 10.1111/j.1524-4733.2005.04054.x

[31] Gjersing L, Caplehorn JR, Clausen T (2010) Cross-cultural adaptation of research instruments: language, setting, time and statistical considerations. BMC Med Res Methodol 10:1–10 20144247 10.1186/1471-2288-10-13 PMC2831007

[32] Alhussein G, Hadjileontiadis L (2022) Digital health technologies for long-term self-management of osteoporosis: systematic review and meta-analysis. JMIR Mhealth Uhealth 10(4):e32557 35451968 10.2196/32557 PMC9073608

[33] Zhou L et al (2019) The mHealth app usability questionnaire (MAUQ): development and validation study. JMIR Mhealth Uhealth 7(4):e11500 30973342 10.2196/11500 PMC6482399

[34] Azimi P et al (2014) An outcome measure of functionality and quality of life in Iranian women with osteoporotic vertebral fractures: a validation study of the QUALEFFO-41. J Orthop Sci 19:860–867 25069808 10.1007/s00776-014-0609-0

[35] Nematollahi M, Eslami AA (2018) Development and validation of social cognitive theory based questionnaire for physical activity to preventing osteoporosis (PAQ-SCT). Iran J Psychiatry Behav Sci. 10.5812/ijpbs.12662

[36] Jeihooni AK et al (2016) Application of the health belief model and social cognitive theory for osteoporosis preventive nutritional behaviors in a sample of Iranian women. Iran J Nurs Midwifery Res 21(2):131–141 27095985 10.4103/1735-9066.178231 PMC4815367

[37] Mustafa N et al (2021) Malay version of the mHealth app usability questionnaire (M-MAUQ): translation, adaptation, and validation study. JMIR Mhealth Uhealth 9(2):e24457 33538704 10.2196/24457 PMC7894394

[38] Khademi K et al (2024) Psychometric validation of the Persian version of Multidimensional Health Locus of Control Scale (MHLC‐C) for menopausal women. Adv Public Health. 10.1155/2024/4667727

[39] Hoque R, Sorwar G (2017) Understanding factors influencing the adoption of mHealth by the elderly: an extension of the UTAUT model. Int J Med Inform 101:75–84 28347450 10.1016/j.ijmedinf.2017.02.002

[40] Venkatesh V, Thong JY, Xu X (2012) Consumer acceptance and use of information technology: extending the Unified Theory of Acceptance and Use of Technology1. MIS Q 36(1):157–178

[41] Bahadori F et al (2024) Information and communication technology adoption strategies among Iranian older adults: a qualitative evaluation. Gerontol Geriatr Med 10:23337214241246315 38633750 10.1177/23337214241246315 PMC11022677

[42] Butler S et al (2022) Effectiveness of eHealth and mHealth interventions supporting children and young people living with juvenile idiopathic arthritis: systematic review and meta-analysis. J Med Internet Res 24(2):e30457 35107431 10.2196/30457 PMC8851322

[43] Harrington CN, Ruzic L, Sanford JA (2017) Universally accessible mHealth apps for older adults: towards increasing adoption and sustained engagement in Universal Access in Human–Computer Interaction. Human and technological environments: 11th International Conference, UAHCI 2017, Held as Part of HCI International 2017, Vancouver, BC, Canada, July 9–14, 2017, Proceedings, Part III 11. Springer

[44] Ghelichkhani F et al (2021) Self-efficacy of osteoporosis preventive behaviors and its predictors in Iranian adolescents. Int J Adolesc Med Health 33(1):20180038 10.1515/ijamh-2018-0038 30352030

[45] Abu Seman S (2020) Willingness to Pay for mHealth Application: The Relationship Between Attitude. Universiti Sains Malaysia, Social Influence and Self-Efficacy with the Moderating Effect of Initial Trust

[46] Darabi F, Ziapour A, Ahmadinia H (2025) Digital health literacy and sociodemographic factors among students in western Iran: a cross-sectional study. BMC Med Educ 25(1):206 39920649 10.1186/s12909-025-06774-y PMC11806557

[47] Norman CD, Skinner HA (2006) EHealth literacy: essential skills for consumer health in a networked world. J Med Internet Res 8(2):e506 10.2196/jmir.8.2.e9 PMC1550701 16867972

[48] Abdipour N, Rakhshanderou S, Ghaffari M (2024) Validity and reliability of the TechPH scale in assessing Iranian older adults’ attitudes toward technology. BMC Geriatr 24(1):907 39501176 10.1186/s12877-024-05502-3 PMC11536867

[49] Koo JH, Park YH, Kang DR (2023) Factors predicting older people’s acceptance of a personalized health care service app and the effect of chronic disease: cross-sectional questionnaire study. JMIR Aging 6(1):e41429 37342076 10.2196/41429 PMC10334719

[50] DeVellis RF, Thorpe CT (2021) Scale development: Theory and applications. Sage publications

