# Supplementary material for: Validation of an mHealth adoption questionnaire for osteoporosis management in Iranian older adults at risk
Source: Arch Osteoporos. 2026 Jul 22;21(1):103. doi: 10.1007/s11657-026-01741-6 (PMC13391763; doi:10.1007/s11657-026-01741-6)
Supplement: Supplementary file 1 — (DOCX 33.2 KB) [file 11657_2026_1741_MOESM1_ESM.docx]

**Appendix A — Table A: Full Questionnaire**

**Table A**. Components of research questionnaire on Factors influencing mHealth tech adoption for osteoporosis management in Iranian adults 50+.

| **NO** | **Items** |
| --- | --- |
| **Performance Expectancy** | 1. Mobile Health (health apps) service can provide me with timely medical information services. |
|  | 2. The mobile health (health apps) service provides me with valuable information resources. |
|  | 3. Mobile Health can reduce my queuing and registration time and improve the efficiency of seeing a doctor. |
|  | 4. Mobile Health has fewer time and space constraints, which increases the convenience of life. |
|  | 5. Overall, mobile Health is helpful to my life. |
| **Effort Expectancy** | 6. Learning how to use the mobile Health (health apps) service is easy for me. |
|  | 7. It is easy for me to become skilled at using the mobile Health (health apps) service. |
|  | 8. I can independently operate a smartphone to obtain mHealth services. |
| **Facilitating Condition** | 9. I have resources( Financial)necessary to use mHealth service. |
|  | 10. I have the knowledge necessary to use the mHealth service. |
|  | 11. I can get help from others when I have difficulties using m-health. |
| **Social Influence** | 12. People who can influence my behavior think I should use the m-Health service. |
|  | 13. People important to me think I should use m-Health service. |
|  | 14. People around me encourage me to use m-Health service. |
|  | 15. People in my society who use mHealth service have more prestigious than those who do not. |
| **Perceived Susceptibility** | 16. I think I have a high risk to get serious osteoporosis |
|  | 17. I feel more vulnerable than others. |
|  | 18. I am at risk of being infected by the osteoporosis. |
|  | 19. It is likely that I would suffer from osteoporosis. |
| **Perceived Severity** | 20. If I were infected by osteoporosis., it would have important health consequences for me. |
|  | 21. If I were affected by osteoporosis., my health would be severely affected. |
|  | 22. If I were affected by osteoporosis, my health would be significantly reduced. |
|  | 23. If I get a serious disease, it will change my whole life. |
|  | 24. I would be afraid to get serious illness. |
| **Technology Anxiety** | 25. Using m-health services make me worried. |
|  | 26. Using mHealth services may make me feel uneasy and confused. |
|  | 27. I hesitate to adopt m-Health service due to being afraid of making a mistake |
|  | 28. Using m-Health service intimidates me |
|  | 29. I have no confidence in my ability to learn a new technology |
|  | 30. It scares me to think that I could lose a lot of information using the smartphone applications by hitting the wrong key. |
|  | 31. I hesitate to use the smartphone applications for fear of making mistakes I cannot correct. |
| **Digital Literacy** | 32. I can use my smart phone to adjust the screen brightness. |
|  | 33. I can use my smart phone to connect to a Wi-Fi network. |
|  | 34. I can use my smart phone to open email & send email. |
|  | 35. I can use my smart phone to view pictures sent by email. |
|  | 36. I can transfer information (files such as music, pictures, and documents) on my computer to my mobile device and reverse. |
|  | 37. I can store information with a service that lets me view my files from anywhere (e.g., Dropbox, Google Drive, Microsoft OneDrive). |
|  | 38. I can find information about local community resources on the Internet. |
|  | 39. I can find health information on the Internet. |
|  | 40. I can watch movies and videos. |
|  | 41. I can read a book. |
|  | 42. I can update games and other applications. |
| **Self-Efficacy** | 43. I can learn how to use mHealth. |
|  | 44. I am sure that I could use such an app. |
|  | 45. I can meet my medical needs through mHealth. |
|  | 46. I’m confident in being able to use mHealth independently. |
|  | 47. I do not feel comfortable using health apps. |
| **Health Status** | 48 I have no problems in walking about.  I have slight problems in walking about.  I have moderate problems in walking about.  I have severe problems in walking about.  I am unable to walk about. |
|  | 49. I have no problems washing or dressing myself.  I have slight problems washing or dressing myself.  I have moderate problems washing or dressing myself.  I have severe problems washing or dressing myself.  I am unable to wash or dress myself. |
|  | 50. I have no problems doing my usual activities.  I have slight problems doing my usual activities.  I have moderate problems doing my usual activities.  I have severe problems doing my usual activities.  I am unable to do my usual activities. |
|  | 51. I have no pain or discomfort.  I have slight pain or discomfort.  I have moderate pain or discomfort.  I have severe pain or discomfort.  I have extreme pain or discomfort. |
|  | 52. I am not anxious or depressed.  I am slightly anxious or depressed.  I am moderately anxious or depressed.  I am severely anxious or depressed.  I am extremely anxious or depressed. |
| **Intentions to Adopt** | 53. When I have related needs, I will choose to use mHealth. |
|  | 54. If mHealth brings convenience to me, I’m willing to continue using it. |
|  | 55. I’m willing to understand or use mHealth. |
|  | 56. I’m willing to use mHealth when I face some diseases or health problems. |
|  | 57. I plan to use mHealth services regularly. |

**Appendix B**: Table B. Relevance and Agreement of the Instrument Items

**Table 2. Relevance and Agreement of the Instrument Items**

| Item | Relevance | | Clarity | | Results |
| --- | --- | --- | --- | --- | --- |
|  | **CVI** | **Kappa** | **CVI** | **Kappa** |  |
| Performance Expectancy 1 | 0.83 | 0.82 | 0.83 | 0.82 | Validated |
| Performance Expectancy 2 | 1.00 | 1.00 | 1.00 | 1.00 | Validated |
| Performance Expectancy 3 | 1.00 | 1.00 | 0.80 | 0.76 | Validated |
| Performance Expectancy 4 | 0.83 | 0.82 | 0.67 | 0.56 | Validated |
| Performance Expectancy 5 | 1.00 | 1.00 | 1.00 | 1.00 | Validated |
| Effort Expectancy 1 | 0.83 | 0.82 | 0.83 | 0.82 | Validated |
| Effort Expectancy 2 | 0.50 | 0.27 | 0.33 | 0.13 | Excluded |
| Effort Expectancy 3 | 0.83 | 0.82 | 0.67 | 0.56 | Validated |
| Effort Expectancy 4 | 0.83 | 0.82 | 0.67 | 0.56 | Validated |
| Effort Expectancy 5 | 0.83 | 0.82 | 0.83 | 0.82 | Validated |
| Facilitating Condition 1 | 0.67 | 0.67 | 0.50 | 0.50 | Excluded |
| Facilitating Condition 2 | 0.83 | 0.83 | 0.83 | 0.83 | Validated |
| Facilitating Condition 3 | 0.50 | 0.50 | 0.50 | 0.50 | Excluded |
| Facilitating Condition 4 | 0.80 | 0.80 | 0.80 | 0.80 | Validated |
| Social Influence 1 | 0.83 | 0.83 | 0.67 | 0.67 | Validated |
| Social Influence 2 | 1.00 | 1.00 | 0.83 | 0.83 | Validated |
| Social Influence 3 | 0.83 | 0.83 | 1.00 | 1.00 | Validated |
| Social Influence 4 | 1.00 | 1.00 | 0.67 | 0.67 | Validated |
| Social Influence 5 | 0.67 | 0.67 | 0.83 | 0.83 | Excluded |
| Perceived susceptibility 1 | 1.00 | 1.00 | 0.50 | 0.50 | Validated |
| Perceived susceptibility 2 | 1.00 | 1.00 | 0.50 | 0.50 | Validated |
| Perceived susceptibility 3 | 0.67 | 0.67 | 0.50 | 0.50 | Excluded |
| Perceived susceptibility 4 | 0.83 | 0.83 | 0.83 | 0.83 | Validated |
| Perceived susceptibility 5 | 0.33 | 0.33 | 0.50 | 0.50 | Excluded |
| Perceived severity 1 | 1.00 | 1.00 | 0.50 | 0.50 | Validated |
| Perceived severity 2 | 1.00 | 1.00 | 0.60 | 0.60 | Validated |
| Perceived severity 3 | 1.00 | 1.00 | 0.50 | 0.50 | Validated |
| Perceived severity 4 | 1.00 | 1.00 | 1.00 | 1.00 | Validated |
| Perceived severity 5 | 1.00 | 1.00 | 1.00 | 1.00 | Validated |
| Digital literacy 1 | 1.00 | 1.00 | 1.00 | 1.00 | Validated |
| Digital literacy 2 | 1.00 | 1.00 | 1.00 | 1.00 | Validated |
| Digital literacy 3 | 1.00 | 1.00 | 0.80 | 0.80 | Validated |
| Digital literacy 4 | 1.00 | 1.00 | 0.80 | 0.80 | Validated |
| Digital literacy 5 | 1.00 | 1.00 | 1.00 | 1.00 | Validated |
| Digital literacy 6 | 0.83 | 0.83 | 0.83 | 0.83 | Validated |
| Digital literacy 7 | 0.83 | 0.83 | 1.00 | 1.00 | Validated |
| Digital literacy 8 | 1.00 | 1.00 | 1.00 | 1.00 | Validated |
| Digital literacy 9 | 1.00 | 1.00 | 1.00 | 1.00 | Validated |
| Digital literacy 10 | 1.00 | 1.00 | 1.00 | 1.00 | Validated |
| Digital literacy 11 | 0.83 | 0.83 | 1.00 | 1.00 | Validated |
| Digital literacy 12 | 0.67 | 0.67 | 1.00 | 1.00 | Excluded |
| Technology Anxiety 1 | 0.67 | 0.67 | 1.20 | 1.20 | Excluded |
| Technology Anxiety 2 | 1.00 | 1.00 | 1.00 | 1.00 | Validated |
| Technology Anxiety 3 | 0.67 | 0.67 | 0.80 | 0.80 | Validated |
| Technology Anxiety 4 | 0.83 | 0.83 | 0.60 | 0.60 | Validated |
| Technology Anxiety 5 | 0.67 | 0.67 | 0.83 | 0.83 | Excluded |
| Technology Anxiety 6 | 1.00 | 1.00 | 1.00 | 1.00 | Validated |
| Technology Anxiety 7 | 1.00 | 1.00 | 0.83 | 0.83 | Validated |
| Technology Anxiety 8 | 0.83 | 0.83 | 1.00 | 1.00 | Validated |
| Technology Anxiety 9 | 0.83 | 0.83 | 1.00 | 1.00 | Validated |
| Technology Anxiety 10 | 0.83 | 0.83 | 1.00 | 1.00 | Validated |
| Technology Anxiety 11 | 0.67 | 0.67 | 0.83 | 0.83 | Excluded |
| Self-efficacy 1 | 0.83 | 0.83 | 1.00 | 1.00 | Validated |
| Self-efficacy 2 | 0.83 | 0.83 | 0.67 | 0.67 | Validated |
| Self-efficacy 3 | 0.67 | 0.67 | 1.00 | 1.00 | Excluded |
| Self-efficacy 4 | 0.83 | 0.83 | 1.00 | 1.00 | Validated |
| Self-efficacy 5 | 0.83 | 0.83 | 1.00 | 1.00 | Validated |
| Self-efficacy 6 | 0.67 | 0.67 | 0.80 | 0.80 | Excluded |
| Self-efficacy 7 | 0.80 | 0.80 | 1.00 | 1.00 | Validated |
| Self-efficacy 8 | 0.83 | 0.83 | 0.50 | 0.50 | Validated |
| Self-efficacy 9 | 0.67 | 0.67 | 0.83 | 0.83 | Excluded |
| Health status 1 | 1.00 | 1.00 | 0.80 | 0.80 | Validated |
| Health status 2 | 1.00 | 1.00 | 1.00 | 1.00 | Validated |
| Health status 3 | 1.00 | 1.00 | 0.83 | 0.83 | Validated |
| Health status 4 | 0.75 | 0.75 | 1.00 | 1.00 | Validated |
| Health status 5 | 0.80 | 0.80 | 1.00 | 1.00 | Validated |
| Health status 6 | 0.80 | 0.80 | 0.83 | 0.83 | Validated |
| Health status 7 | 0.80 | 0.80 | 0.80 | 0.80 | Validated |
| Health status 8 | 0.60 | 0.60 | 0.67 | 0.67 | Excluded |
| Health status 9 | 0.60 | 0.60 | 0.83 | 0.83 | Excluded |
| Health status 10 | 0.80 | 0.80 | 1.00 | 1.00 | Validated |
| Health status 11 | 1.00 | 1.00 | nan | nan | Validated |
| Health status 12 | 1.00 | 1.00 | 1.00 | 1.00 | Validated |
| Health status 13 | 1.00 | 1.00 | 1.00 | 1.00 | Validated |
| Health status 14 | 1.00 | 1.00 | 1.00 | 1.00 | Validated |
| Health status 15 | 1.00 | 1.00 | 1.00 | 1.00 | Validated |
| intentions to Adopt 1 | 1.00 | 1.00 | 0.67 | 0.67 | Validated |
| intentions to Adopt 2 | 1.00 | 1.00 | 0.83 | 0.83 | Validated |
| intentions to Adopt 3 | 0.83 | 0.83 | 0.83 | 0.83 | Validated |
| intentions to Adopt 4 | 0.83 | 0.83 | 0.83 | 0.83 | Validated |
| intentions to Adopt 5 | 0.83 | 0.83 | 0.83 | 0.83 | Validated |

**Appendix C: Table C: Item reduction by construct across validation stages**

| **Construct** | **Initial pool** | **Removed after CVI/Kappa** | **Removed after Cognitive debriefing** | **Removed after EFA** | **Final** |
| --- | --- | --- | --- | --- | --- |
| Performance Expectancy (PE) | 5 | 0 | 0 | 0 | **5** |
| Effort Expectancy (EE) | 5 | 2 | 0 | 0 | **3** |
| Facilitating Conditions (FC) | 4 | 1 | 0 | 0 | **3** |
| Social Influence (SI) | 5 | 1 | 0 | 0 | **4** |
| Perceived Susceptibility (PSU) | 5 | 1 | 0 | 0 | **4** |
| Perceived Severity (PSE) | 5 | 0 | 0 | 0 | **5** |
| Digital Literacy (DL) | 12 | 1 | 0 | 2 | **9** |
| Technology Anxiety (TA) | 11 | 4 | 0 | 0 | **7** |
| Self-efficacy (SE) | 9 | 3 | 1 | 1 | **4** |
| Health Status (HS) | 15 | 10 | 0 | 0 | **5** |
| Intentions to Adopt (IA) | 5 | 0 | 0 | 0 | **5** |
| **Total** | **81** | **23** | **1** | **3** | **54** |

**Note:** Columns show the number of items removed at each stage. CVI = content validity index; EFA = exploratory factor analysis.

EFA removed SE5 (factor loading < 0.30) and DL3, DL4 (cross-loading on a second factor, compromising one-dimensionality of the digital literacy construct).

Following confirmatory factor analysis (CFA; n = 500), HS5 was additionally excluded due to a low factor loading (0.343), yielding a final instrument of 54 items.
